# StagX1, an isoquinolinone compound, selectively targets CES1-positive Ewing sarcoma cells as a potential therapeutic agent

**DOI:** 10.1016/j.omton.2026.201321

**Published:** 2026-08-11

**Authors:** Nenggang Zhang, Scott R. Gilbertson, Feng Li, Debananda Pati

**Affiliations:** 1 Texas Children’s Cancer and Hematology Center, Houston, TX 77030, USA; 2 Department of Pediatrics, Baylor College of Medicine, Houston, TX 77030, USA; 3 Center for Drug Discovery, Baylor College of Medicine, Houston, TX 77030, USA; 4 Department of Pathology and Immunology, Baylor College of Medicine, Houston, TX 77030, USA; 5 NMR and Drug Metabolism Core, Advance Technology Core, Baylor College of Medicine, Houston, TX 77030, USA; 6 Department of Chemistry, University of Houston, Houston TX 77204, USA

**Keywords:** StagX1, SID7969543, StagX1-acid, CES1, Ewing sarcoma, drug metabolism, pharmacokinetics, apoptosis

## Abstract

StagX1 {ethyl 2-[[2-[2-[(2,3-dihydro-1,4-benzodioxin-6-yl)amino]-2-oxoethyl]-1,2-dihydro-1-oxo-5-isoquinolinyl]oxy]propanoate} is a derivative of isoquinolinone, possessing an ethyl propionate. StagX1 exhibits growth-inhibitory activity in multiple Ewing sarcoma cell lines. To advance StagX1 as a potential lead, we conducted experiments to examine its metabolism and stability in tissue culture media, plasma, liver microsomes, cells and mice. Our studies demonstrate that StagX1 is metabolically unstable and undergoes rapid hydrolysis to its corresponding acid metabolite (StagX1-acid) through cleavage of the ethyl ester group. We identified carboxylesterase 1 (CES1) as the primary enzyme responsible for this conversion. Notably, cells expressing CES1 are sensitive to StagX1, whereas CES1-deficient cells show minimal response, indicating that metabolic activation is required for its activity. In contrast, StagX1-acid is metabolically stable. These findings suggest that StagX1 functions as a prodrug that is enzymatically converted to its active metabolite, StagX1-acid, within cells. This metabolic conversion likely underlies its mechanism of action and contributes to its selective anticancer activity in Ewing sarcoma. Our findings provide insight into the metabolism of StagX1 and the role of CES1 in mediating its effects and demonstrate that StagX1 is a promising compound with growth inhibitory effects in CES1 positive Ewing sarcoma cells.

## Graphical abstract

**Figure:**
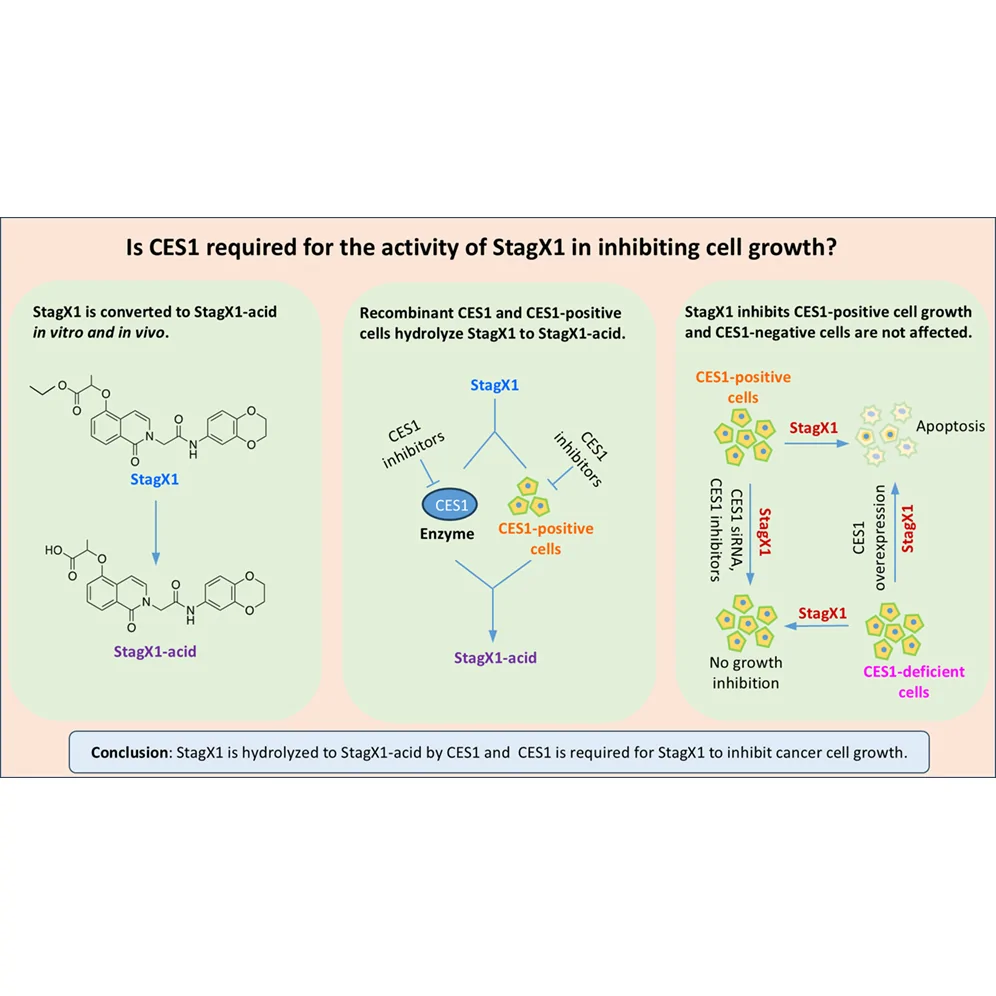

## Teaser

Zhang and colleagues show that StagX1 selectively inhibits CES1-expressing Ewing sarcoma cells. They demonstrate that CES1 hydrolyzes StagX1 into its active acid metabolite, establishing CES1-mediated metabolism as essential for its antitumor activity. These findings identify StagX1 as a promising lead for targeted therapy in CES1-positive Ewing sarcoma.

## Introduction

Ewing sarcoma (ES) is a rare but aggressive pediatric cancer that has poor prognosis and is inadequately served by current therapies.^1^^,^^2^ In the United States, ES has an annual incidence of 2.93 children per million, with approximately 200–250 children and adolescents diagnosed each year. Despite its rarity, ES is responsible for a significant number of pediatric cancer deaths, especially among patients with advanced and refractory tumors. Current treatment options for relapsed and refractory ES are limited, and there have been no new FDA-approved drugs for ES in the past 60 years.

StagX1, also known as SID7969543, was initially identified as a lead compound through a high throughput screen aimed at selectively inhibiting the growth of ES cell lines harboring *STAG2* mutation.^3^ *STAG2* is a genomic alteration present in approximately 14%–21.5% of ES patients and often associated with increased metastatic risk and poor clinical outcomes. Although originally thought to function through synthetic lethality with STAG2 loss, subsequent investigations revealed that the selective cytotoxicity of StagX1 toward STAG2-mutant ES cells is not dependent on STAG2 status. Beyond its activity in ES, recent studies demonstrate that StagX1 also reduces the viability of a subset of mixed lineage leukemia (MLL) cell lines, particularly those harboring MLL/*KMT2A*-rearrangement or *CALM-AF10* translocations.^4^ Of note, StagX1 was previously reported as a putative inhibitor of the nuclear receptor NR5A1 (SF-1),^5^ a transcription factor involved in sex determination and adrenal development, and was shown to exert cytotoxic effects on adrenocortical carcinoma cell lines in a NR5A1-dependent manner.^6^ However, StagX1-mediated inhibition of ES and MLL cell growth appears to be independent of NR5A1 expression,^3^^,^^4^ suggesting alternative molecular targets and mechanisms of action in these malignancies.

In this study, we identify carboxylesterase 1 (CES1) as a key molecular target associated with StagX1 activity. To advance StagX1 as a potential drug lead, we performed comprehensive *in vitro* and *in vivo* analyses examining its metabolic stability, pharmacokinetics (PK) and the biological effects of its primary metabolites. Our data demonstrate that StagX1 undergoes rapid hydrolysis of its ethyl ester, generating StagX1-acid *in vitro and in vivo*. We further established that CES1 is responsible for this conversion and that pharmacologic inhibition of CES1 not only blocks the formation of StagX1-acid but also eliminates the growth-inhibitory effects of StagX1. Notably, we find that StagX1 sensitivity in ES cells correlates strongly with CES1 expression: cell lines responsive to StagX1 are CES1-positive, whereas insensitive lines lack CES1 activity. These findings highlight CES1 as a critical determinant of StagX1 metabolism and anti-tumor efficacy.

## Results

### StagX1 induces apoptosis in a subset of ES cell lines

**Figure 1:**
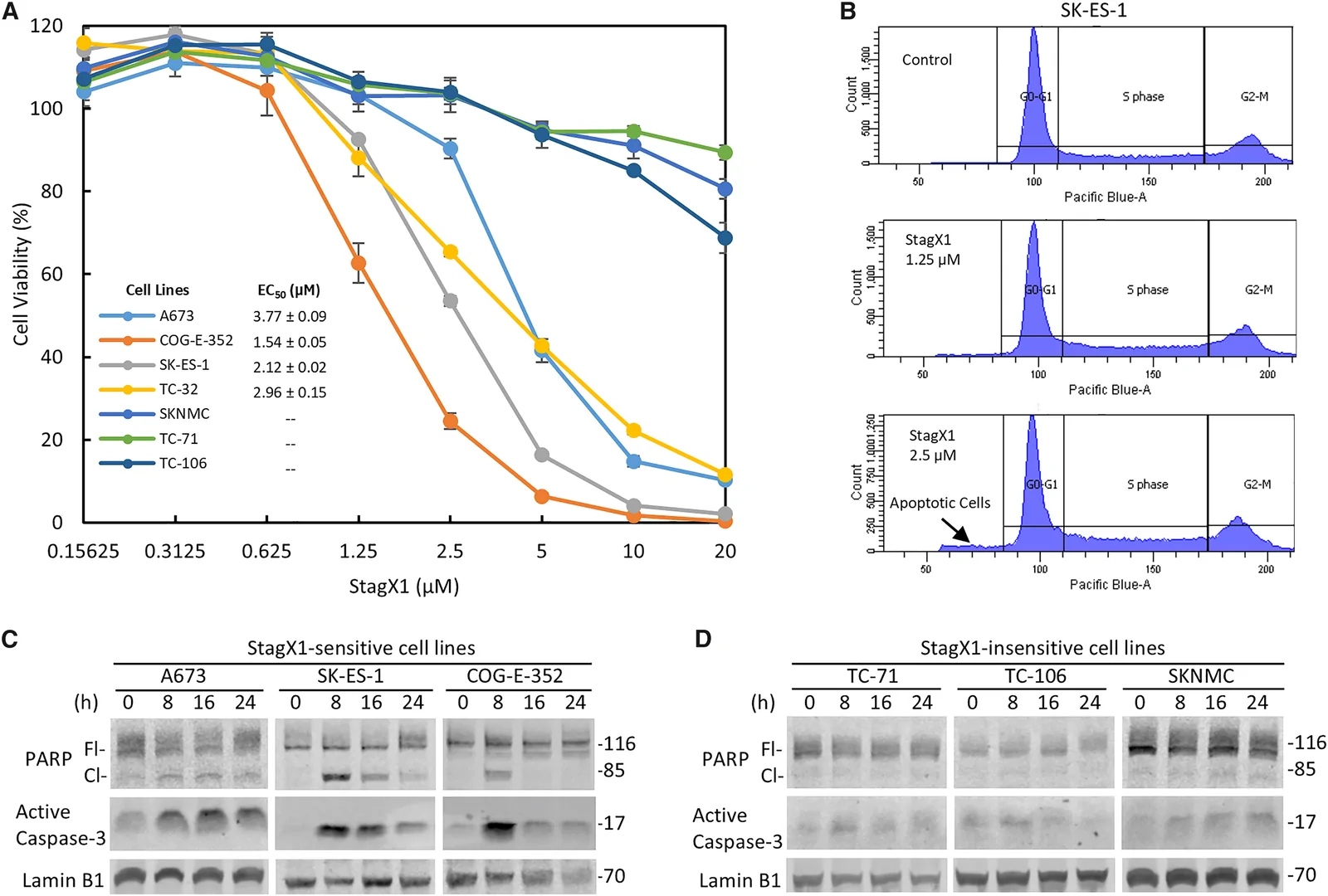
StagX1 induces apoptosis in a subset of Ewing sarcoma cell lines (A) Seven ES cells were treated with StagX1 for 3 days and cell viability were determined. StagX1 inhibited cell growth of 4 out of 7 ES cell lines. The data are presented as mean ± SD with *n* = 3. (B) SK-ES-1 cells were treated with various concentrations of StagX1 for 24 h. Cell cycle was analyzed with FACS. StagX1 did not affect cell cycle. Apoptotic cells were found in treatment with 2.5 μM StagX1. (C and D) StagX1-sensitive (C) and -insensitive (D) ES cell lines were treated with 10 μM of StagX1 for various times before protein samples were prepared. Immunoblotting analysis was performed using antibodies against PARP and caspase-3 to detect PARP cleavage and the active caspase-3 band (17 KDa), with Lamin B1 serving as the loading control. Cleavage of PARP and caspase-3 was observed only in the StagX1-sensitive ES cell lines, demonstrating that StagX1 induces apoptosis selectively in responsive cells.

To evaluate the effects of StagX1 on ES cell growth, we examined seven ES cell lines: A673, COG-E-352, SK-ES-1, SKNMC, TC-32, TC-71, and TC-106. Cell viability assays revealed that four of seven cell lines (A673, COG-E-352, SK-ES-1, and TC-32) were sensitive to StagX1 treatment, whereas the remaining three (SKNMC, TC-71, and TC-106) were minimally affected (Figure 1A). To determine whether growth inhibition was associated with alterations in cell cycle progression, we analyzed cell cycle distribution in SK-ES-1 cells following StagX1 treatment. No significant changes in cell cycle phase distribution were observed, except for an increased apoptotic population at the highest dose treated (2.5 μM) (Figure 1B). To assess activation of apoptotic pathways, we performed immunoblotting. StagX1 treatment resulted in robust activation of caspase-3 and cleavage of PARP in StagX1-sensitive cell lines A673, SK-ES-1, and COG-E-352 (Figure 1C), consistent with the activation of apoptosis cascade. In contrast, no detectable active caspase-3 or PARP cleavage was observed in StagX1-insensitive cell lines (SKNMC, TC-71, and TC-106) (Figure 1D), indicating that apoptosis is selectively induced in StagX1-sensitive cells. Collectively, these data indicate that StagX1 inhibits ES cell growth primarily through induction of apoptosis in a subset of ES cell lines.

### StagX1 is metabolized to StagX1-acid *in vitro* and *in vivo*

**Figure 2:**
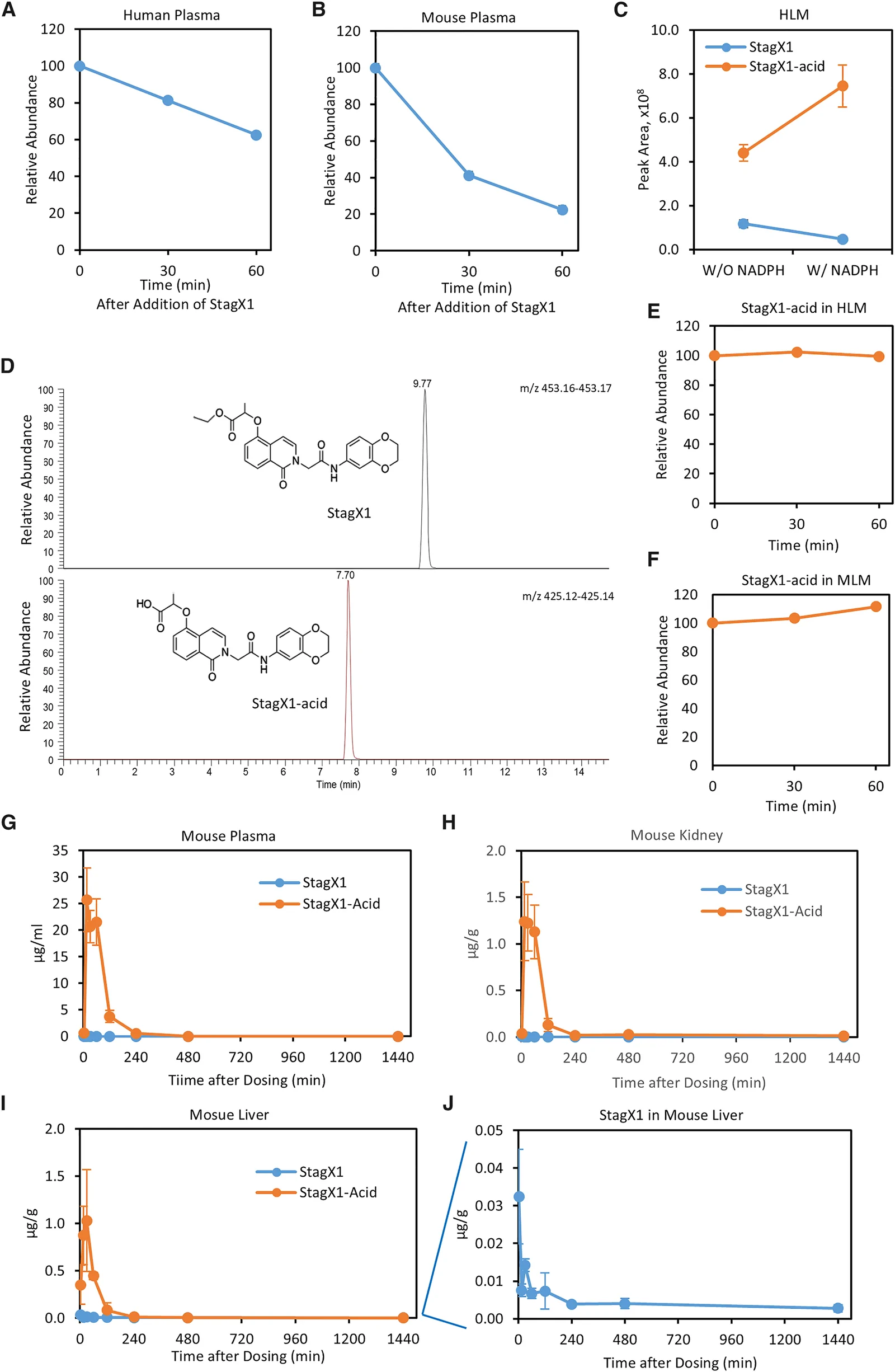
StagX1-acid is a metabolic product of StagX1 (A and B) The relative abundance of StagX1 after incubation with mouse and human plasma and shown as mean of two replicates. (C) The relative abundance of StagX1 and StagX1-acid in human liver microsome (HLM) where StagX1 was incubated without (W/O) or with (W/) NADPH for 30 min. The abundance of StagX1 and StagX1-acid was determined by LC-MS, and the data are presented as mean ± SD with *n* = 3. (D). Chromatograms of StagX1 and StagX1-acid. (E and F) The relative abundance of StagX1-acid after incubation with HLM and mouse liver microsome (MLM) is shown as mean of two replicates. (G–J) The pharmacokinetics (PK) of StagX1 in mice. SCID-beige mice were administered with 450 μg/kg of StagX1 via intraperitoneal injection. Blood, liver, and kidney samples were collected at different time points after injection, and the levels of StagX1 and its metabolic product StagX1-acid were determined by UHPLC-MS. StagX1 and StagX1-acid levels in mouse plasma (G), kidney (H), and liver (I) were measured. StagX1 was undetectable in plasma and kidney samples but was detected at low levels in liver. Data are presented as mean ± SD (*n* = 4).

To investigate the stability of StagX1, we incubated StagX1 with human and mouse plasma as well as human liver microsomes (HLMs). The relative abundance of StagX1 in the solution was determined using liquid chromatography-mass spectrometry (LC-MS). The results showed that StagX1 was reduced by approximately 20% in 30 min and 40% in 1 h in human plasma (Figure 2A). However, the loss of StagX1 was greater in mouse plasma, with approximately 60% reduction in the first 30 min and 80% reduction in 1 h (Figure 2B). As StagX1 is an ester, we hypothesized that it might be hydrolyzed to form an acid. In HLM, we found that StagX1 was converted to an acid (Figure 2C). The hydrolyzed metabolite was named as StagX1-acid, and its chemical structure (2-[[2-[2-[(2,3-dihydro-1,4-benzodioxin-6-yl)amino]-2-oxoethyl]-1,2-dihydro-1-oxo-5-isoquinolinyl]oxy]propanoic acid) was confirmed using synthetic standard compound (Figure 2D) by comparison of the exact mass and retention time.

Although StagX1 is unstable in liver microsomes, its metabolite, StagX1-acid, remained unchanged for at least 60 min (Figures 2E and 2F), indicating that StagX1-acid is stable.

Additionally, we conducted *in vivo* pharmacokinetic studies of StagX1 in mice. SCID-beige mice were administered StagX1 via intraperitoneal injection, and blood, liver, and kidney samples were collected at various time points after injection. LC-MS analysis was used to determine the levels of StagX1 and StagX1-acid in the samples. The data showed that StagX1 was not detectable in either the blood or kidney samples at any of the time points examined (Figures 2G and 2H). Although StagX1 was detected in liver samples (Figure 2I), the levels were extremely low and maintained relatively stable level after the initial spike (Figure 2J). Conversely, the levels of StagX1-acid significantly increased in blood and kidney samples at 15, 30, and 60 min post injection, and undetectable after 4 h (Figures 2G and 2H). The level of StagX1-acid in the liver also increased within 2 h after injection (Figure 2I). Detailed PK parameters are shown in Table S1. The results suggest that StagX1 is rapidly metabolized to StagX1-acid in the blood and liver and is subsequently eliminated by the kidney.

### StagX1 is hydrolyzed by CES1

**Figure 3:**
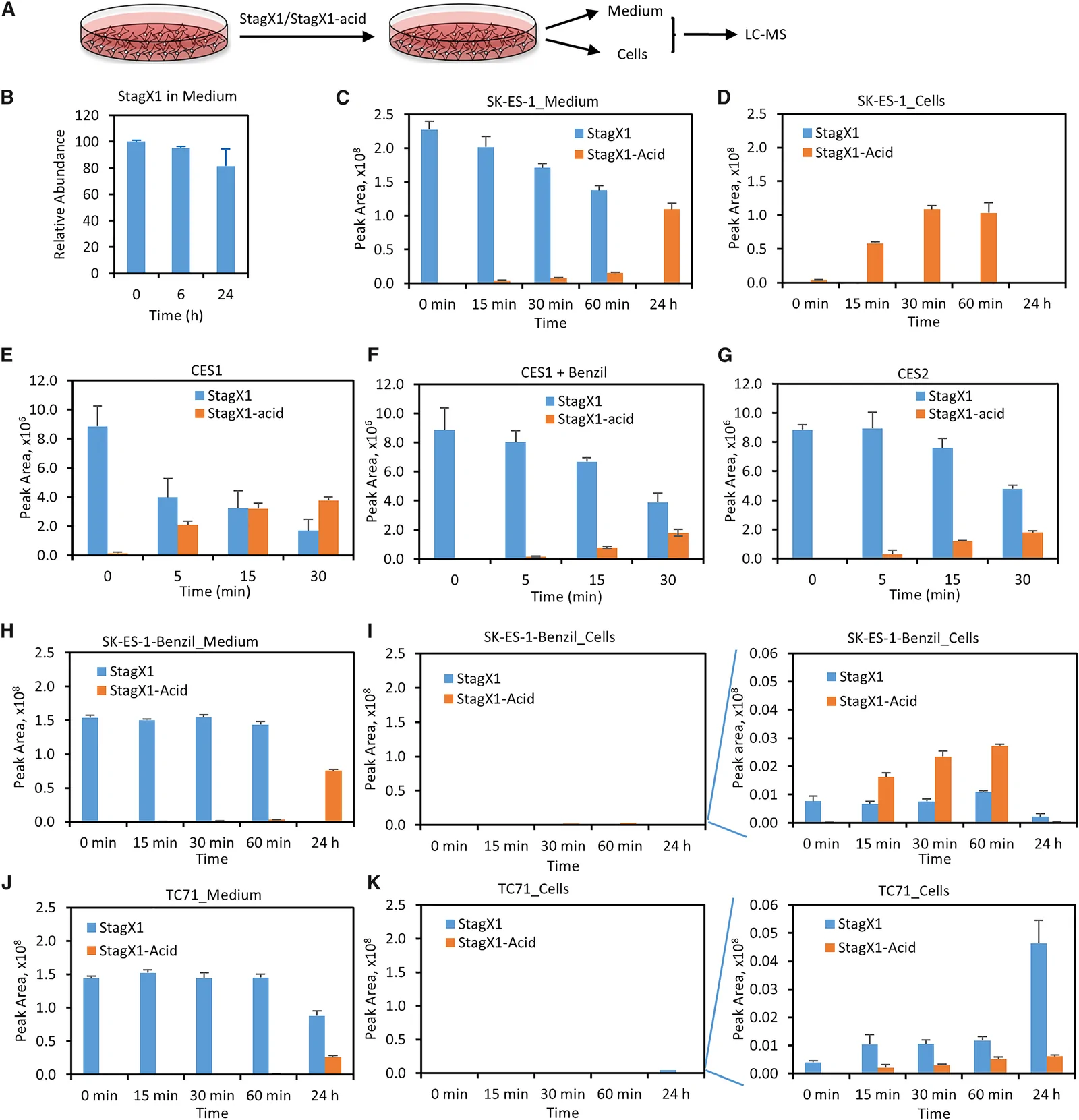
StagX1 is metabolized to StagX1-acid in cells (A) Schematic illustrating the experimental workflow used to analyze StagX1 and StagX1-acid in the culture medium and cells following StagX1 treatment. (B) StagX1 is relatively stable in culture medium (RPMI1640 + 10% FBS). The level of StagX1 was determined after it was incubated in culture medium for 0, 6, and 24 h. (C and D) Following treatment of SK-ES-1 cells with StagX1, the concentration of StagX1 and StagX1 in the cell culture medium (C) and cells (D) were measured. StagX1 was not detected within the cells, however, intracellular StagX1-acid levels increased rapidly, peaking within 60 min, and became undetectable by 24 h (E–G) Hydrolysis of StagX1 by recombinant CES1 and CES2. CES1 efficiently converted StagX1 to StagX1-acid by CES1 (E), whereas benzil inhibited CES1-mediated hydrolysis of StagX1 (F). In contrast, CES2 exhibited substantially lower hydrolytic activity toward StagX1 (G). (H and I) Benzil reduced the conversion of StagX1 to StagX1-acid in SK-ES-1 cells. (J and K) StagX1 was not converted to StagX1-acid in TC-71 cells. Data are presented as mean ± SD (*n* = 3).

To investigate whether the hydrolysis of StagX1 takes place inside cells, we used an ES cell line SK-ES-1, which is sensitive to StagX1 and whose growth can be inhibited by StagX1.^3^ We treated cells with StagX1 and collected samples of both the medium and cells at different times to measure the formation of StagX1-acid (Figure 3A). When StagX1 was present in the tissue culture medium (RPMI1640 + 10% fetal bovine serum [FBS]) without cells, its concentration decreased by 5% every 6 h within 24 h (Figure 3B). However, StagX1 was reduced much more rapidly and disappeared within 24 h in the medium with SK-ES-1 cells (Figure 3C). In contrast, StagX1-acid was increased in the medium (Figure 3C). In SK-ES-1 cells, StagX1 was hardly detectable, whereas StagX1-acid rapidly increased within 30 min (Figure 3D). Surprisingly, StagX1-acid in the cells disappeared at 24 h after StagX1 treatment (Figure 3D), whereas the level of StagX1-acid in the medium was found to be the highest (Figure 3C). These findings suggest that StagX1 is transported into cells and hydrolyzed to StagX1-acid, which is then exported outside the cells.

As StagX1 is an ester, it is likely to undergo hydrolysis by carboxylesterases (CES). To confirm this hypothesis, we conducted an experiment where recombinant human CES1 or CES2 proteins were incubated with StagX1 for varying lengths of time. StagX1 remained stable in the assay buffer in the absence of enzyme, indicating that it does not undergo spontaneous hydrolysis under the assay conditions (Figure S1). The level of StagX1-acid increased in a time-dependent manner following incubation of StagX1 with CES1 (Figure 3E). The production of StagX1-acid was reduced by the CES1 inhibitor, benzil (Figure 3F). However, when StagX1 was mixed with CES2, the formation of StagX1-acid was less and slower than that with CES1 (Figures 3E and 3G). These results suggest that CES1 is predominantly responsible for the hydrolysis of StagX1 to StagX1-acid.

Since CES1 hydrolyzes StagX1 to the acid form *in vitro*, we predict that CES1 is responsible for the hydrolysis of StagX1 inside SK-ES-1 cells. Inhibitors of CES1 should be able to block this hydrolysis. In fact, when the CES1 inhibitor, benzil, was added to SK-ES-1 cells, StagX1-acid formation was inhibited during the first 60 min (Figures 3H and 3I), compared to cells without the CES1 inhibitor (Figures 3C and 3D). Although the conversion of StagX1 to the acid form was slow in the presence of benzil, about 70% of StagX1-acid was still generated at 24 h (Figure 3H, compared with Figure 3C). This suggests that benzil cannot completely inhibit the activity of CES1. Other enzymes or background hydrolysis might be involved in the formation of StagX1-acid.

Furthermore, we conducted experiments to assess the hydrolysis of StagX1 in a different ES cell line, TC-71, which is not sensitive to StagX1, and found that StagX1 has no effect on the growth of these cells.^3^ Interestingly, we observed very little hydrolysis of StagX1 to StagX1-acid in TC-71 cells (Figures 3J and 3K). At 24 h, more than 60% of StagX1 remained in the medium (Figure 3J), and relatively high levels of both StagX1 and StagX1-acid were detected in TC71 cells (Figure 3K right). The efficiency of StagX1 hydrolysis to StagX1-acid in TC-71 cells was much lower than that observed in SK-ES-1 cells (Figures 3D and 3K). The hydrolysis pattern of StagX1 in TC-71 cells (Figures 3J and 3K) closely resembled that observed in benzil-treated SK-ES-1 cells (Figures 3H and 3I). These findings suggest that there is no CES1 activity in TC-71 cells and that other enzymes are inefficient in hydrolyzing StagX1.

### StagX1-acid does not enter cells

**Figure 4:**
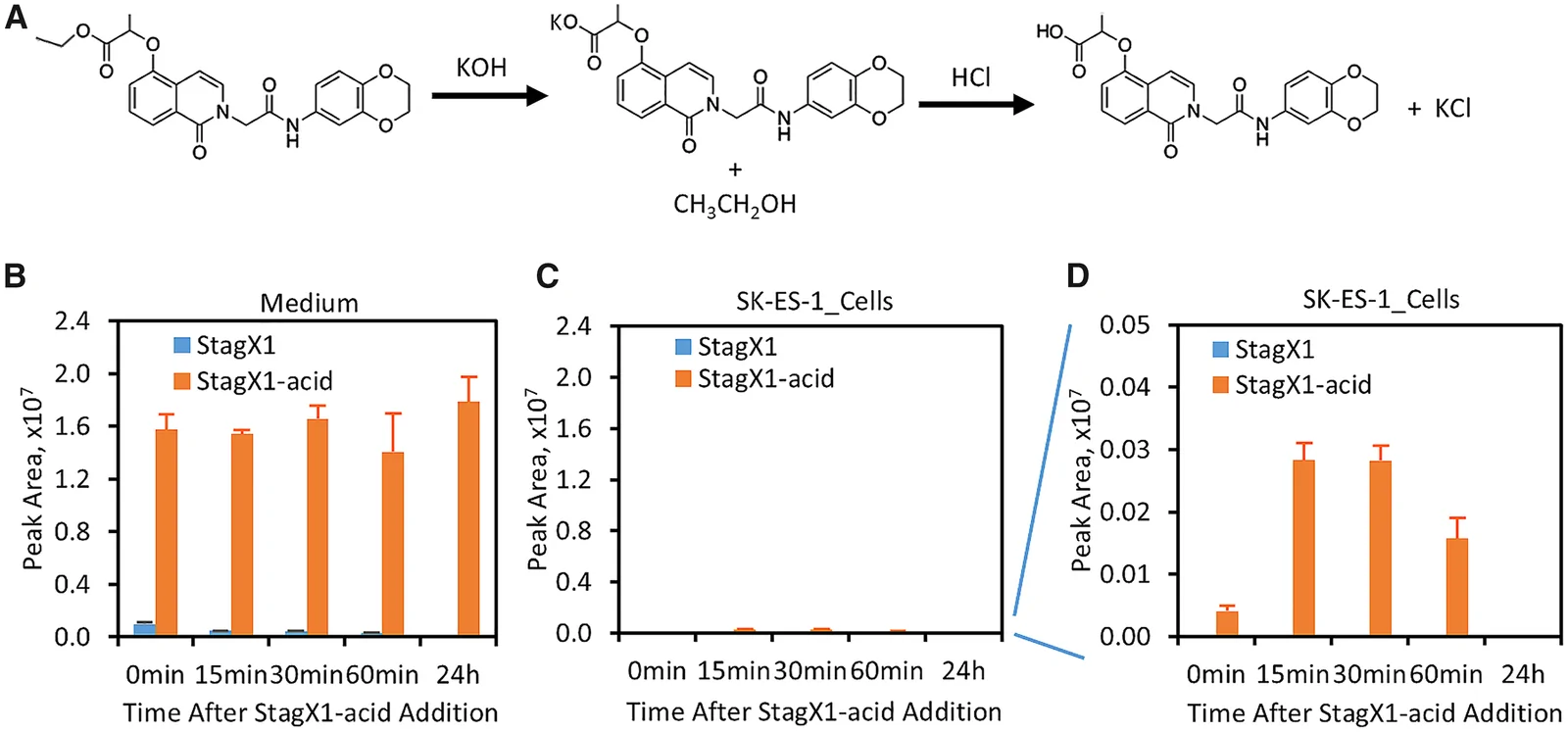
StagX1-acid is prevented from entering the cells (A) To generate StagX1-acid, StagX1 is hydrolyzed using potassium hydroxide (KOH) and hydrochloric acid (HCl). (B–D) After treating SK-ES-1 cells with StagX1-acid for various durations, the levels of StagX1-acid and any residual StagX1 were measured in the medium (B) and the cells (C and D). Data are presented as mean ± SD (*n* = 3).

To assess whether StagX1-acid can enter cells, we synthesized it by hydrolyzing StagX1 with potassium hydroxide (Figure 4A) and treated SK-ES-1 cells with the resulting compound. Medium and cell samples were collected at 0, 15, 30, and 60 min, as well as 24 h post-treatment, and analyzed by LC-MS. StagX1-acid levels in the medium remained consistently high across all time points (Figure 4B), whereas only a small fraction was detected within cells (Figure 4C). These findings suggest that StagX1-acid is largely unable to enter cells.

**Figure 6:**
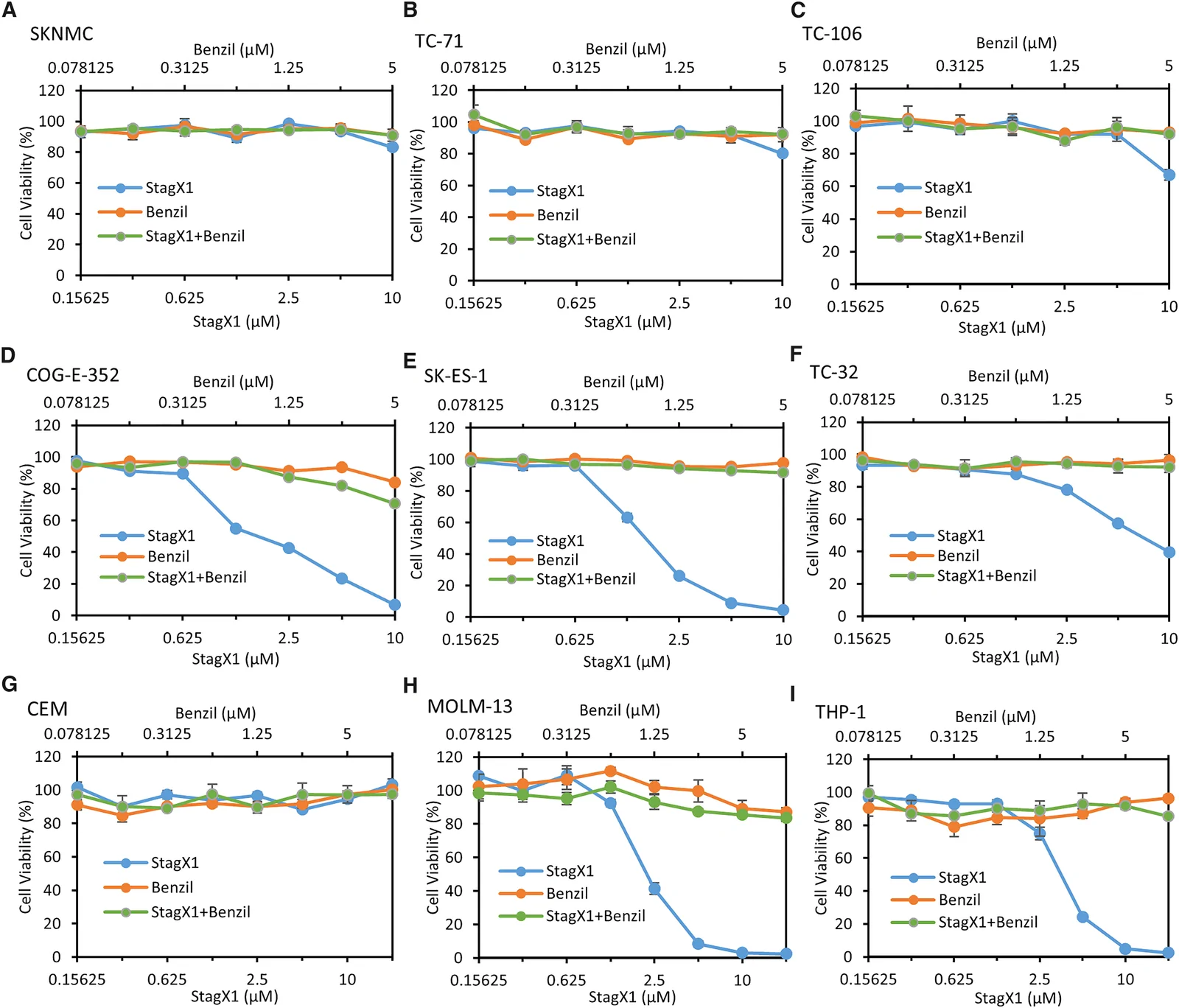
Benzil, a CES inhibitor, blocks the growth-inhibitory effect of StagX1 (A–C) Treatment with benzil, StagX1, or their combination had no effect on the growth of Ewing sarcoma cell lines SKNMC, TC-71, and TC-106. (D–F) Benzil abolished the growth-inhibitory effect of StagX1 in the Ewing sarcoma cell lines COG-E-352, SK-ES-1, and TC-32. (G) Treatment with benzil, StagX1, or their combination had no effect on growth of the acute lymphoblastic leukemia (ALL) cell line CEM. (H and I) Benzil blocked the growth-inhibitory effect of StagX1 in the acute myeloid leukemia (AML) cell lines MOLM-13 and THP-1. Data are presented as mean ± SD (*n* = 3).

We observed a slight increase in StagX1-acid in the medium after 24 h (Figure 4B), which could be due to impurities in the StagX1-acid preparation. Nuclear magnetic resonance (NMR) analysis did not identify the impurity, but LC-MS data showed that about 6% of StagX1 remained in the StagX1-acid preparation (calculated by dividing the relative abundance of StagX1 by that of StagX1-acid at 0 min) (Figure 4B), which may have contributed to the increase in the StagX1-acid in the medium over time. Although we could not detect StagX1 in cells, the early time points showed an inverse correlation between the increase in StagX1-acid inside the cells and the amount of StagX1 in the medium (Figures 4B and 4D). After 24 h, we detected no StagX1 in the medium and no StagX1-acid in the cells (Figures 6B and 6D). This suggests that the StagX1-acid in the cells was derived from the StagX1 in the medium, i.e., StagX1 was imported into the cells and converted to StagX1-acid, which was then exported from the cells back to the medium.

Based on the observed relationship between StagX1 and StagX1-acid in both the medium and cells, we conclude that StagX1 is hydrolyzed to StagX1-acid by CES1 intracellularly, after which the metabolite is subsequently exported.

### CES1 expression levels in cells are positively correlated with their sensitivity to StagX1

In Figure 3, we demonstrated that StagX1 is converted to StagX1-acid by recombinant human CES1 and in StagX1-sensitive SK-ES-1 cells. However, this conversion was inefficient in StagX1-insensitive TC-71 cells (Figure 3J) and was markedly reduced in SK-ES-1 cells in the presence of the CES1 inhibitor benzil (Figure 3H). Together, these findings suggest that CES1 plays a critical role in determining cellular sensitivity to StagX1.

**Figure 5:**
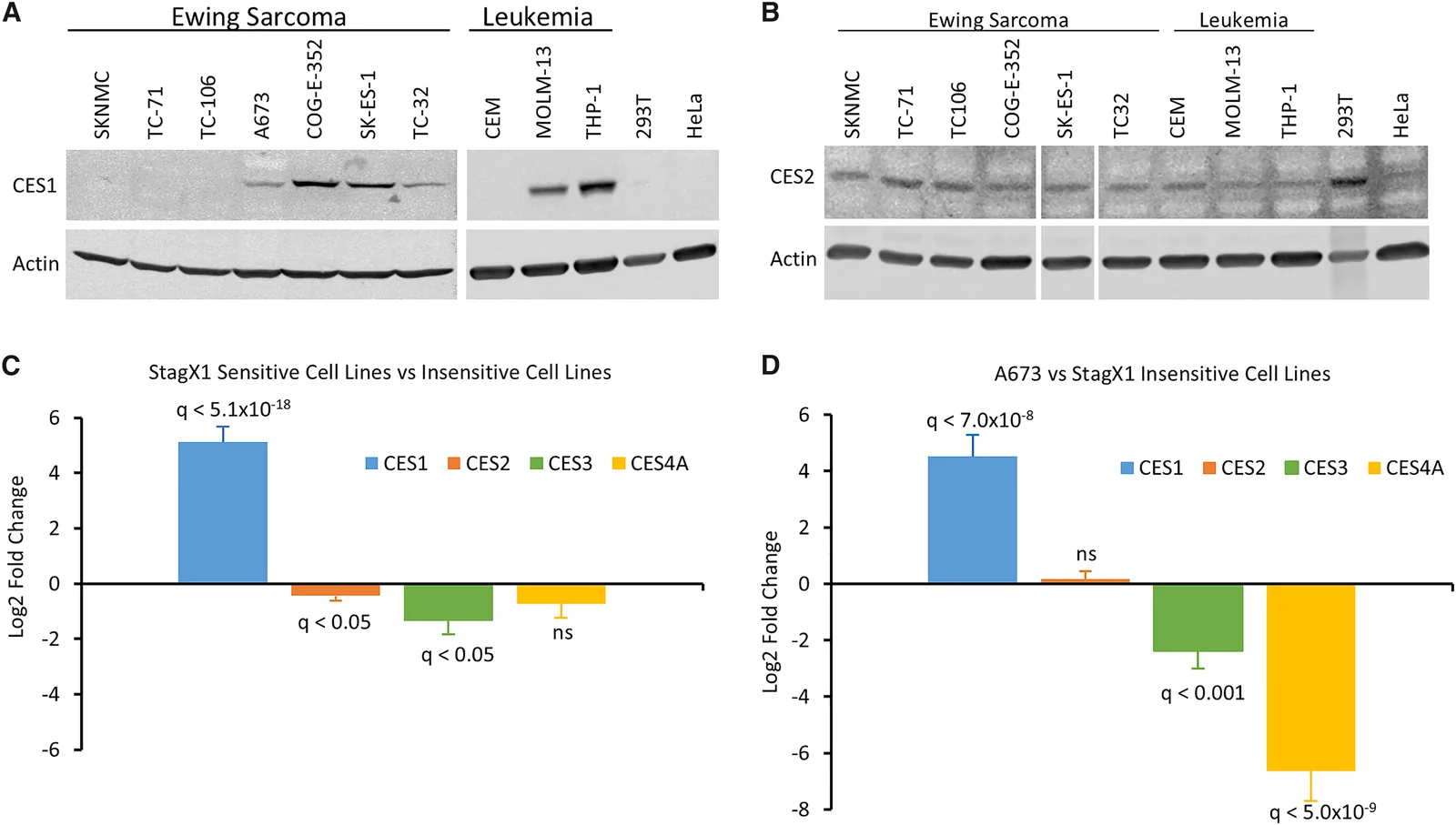
CES1 expression in a panel of cancer cell lines (A and B) The expression of CES1 and CES2 protein was evaluated in a panel of cancer cell lines using immunoblotting. (C) The relative mRNA expression levels of *CES1*, *CES2*, *CES3*, and *CES4A* were determined by RNA-seq in StagX1-sensitive Ewing sarcoma cell lines (COG-E-352, SK-ES-1, and TC-32) and compared with those in StagX1-insensitive cell lines (SKNMC, TC-71, and TC-106). (D) Relative mRNA expression levels of the same CES family members were also compared between the moderately StagX1-sensitive Ewing sarcoma cell line A673 and the StagX1-insensitive cell lines (SKNMC, TC-71, and TC-106). q, false discovery rate (FDR)-adjusted *p* value; ns, not significant. Data are presented as mean ± SD (*n* = 3).

To test this hypothesis, we first examined CES1 protein levels across multiple cell lines (Figure 5A). Immunoblot analysis showed that StagX1-insensitive ES cell lines (SKNMC, TC-71, and TC-106), as well as CEM, 293T, and HeLa cells, had no detectable CES1 expression. In contrast, StagX1-sensitive ES cell lines (A673, COG-E-352, SK-ES-1, and TC-32), along with the leukemia cell lines MOLM-13 and THP-1, exhibited clear CES1 bands. In comparison, CES2 protein levels were relatively similar across all cell lines examined (Figure 5B).

Consistent with these findings, RNA-seq data showed that CES1 expression was significantly higher in StagX1-sensitive ES cell lines (COG-E-352, SK-ES-1, and TC-32) than in StagX1-insensitive lines (SKNMC, TC-71, and TC-106) (Figure 5C). Moreover, CES1 mRNA levels in the moderately StagX1-sensitive cell line A673 were also significantly higher than those in the insensitive lines (Figure 5D).

Taken together, these results suggest that cellular sensitivity to StagX1 positively correlates with CES1 expression levels.

### CES1 inhibitors neutralize the growth-inhibitory effect of StagX1

If conversion of StagX1 to StagX1-acid is required for its growth-inhibitory activity, then inhibition of CES1 should counteract this effect. To test this hypothesis, cells were treated with StagX1 and the CES1 inhibitor benzil, either alone or in combination. Cell viability assays showed that StagX1-insensitive ES cell lines (SKNMC, TC-71, and TC-106) were unaffected by these treatments (Figures 6A–6C). In contrast, StagX1-sensitive ES cell lines (COG-E-352, SK-ES-1, and TC-32) became resistant to StagX1 in the presence of benzil (Figures 6D–6F).

Similar results were observed in leukemia cell lines. The growth-inhibitory effect of StagX1 was abolished by benzil in StagX1-sensitive lines (MOLM-13 and THP-1), whereas the insensitive cell line CEM remained unaffected (Figures 6G–6I). We further evaluated additional CES inhibitors. Phenanthrene-9,10-dione similarly eliminated the inhibitory effect of StagX1 (Figure S2), consistent with the effect of benzil. Other inhibitors, including lovastatin, simvastatin, Tween 20, and NaF, also reduced or attenuated the impact of StagX1 on cell viability (Figure S3).

### Manipulating CES1 levels alters cellular sensitivity to StagX1

**Figure 7:**
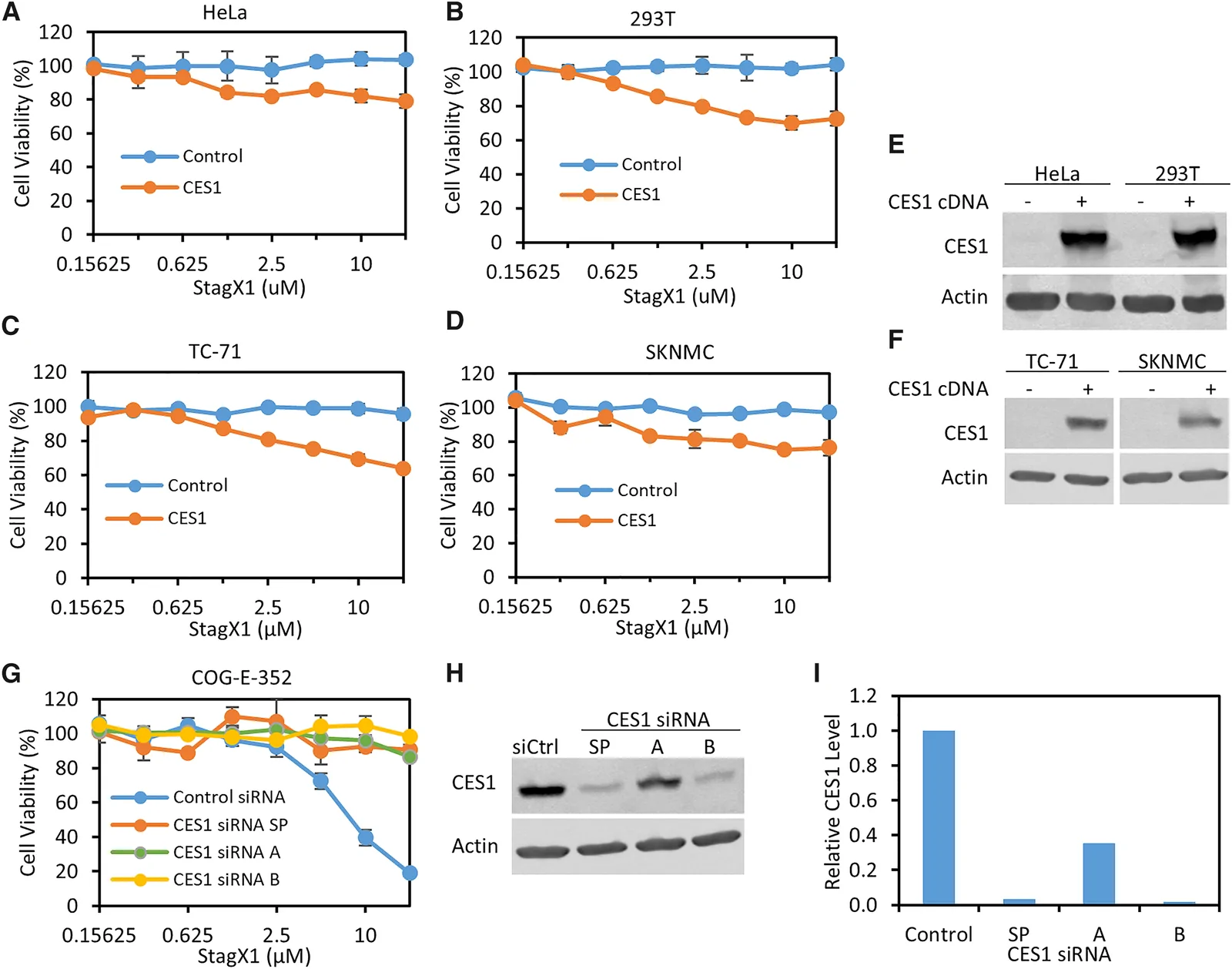
CES1 overexpression sensitizes whereas CES1 knockdown desensitizes cancer cells to StagX1 (A–D) Ectopic overexpression of CES1 in cancer cell lines lacking endogenous CES1 expression sensitizes the cells to StagX1. (E and F) Immunoblotting confirming the CES1 overexpression in HeLa, 293T, TC-71, and SKNMC cells. (G) Knockdown of CES1 by siRNA attenuated the growth-inhibitory effect of StagX1 in COG-E-352 cells. (H) Immunoblotting confirming CES1 knockdown in COG-E-352 following siRNA transfection. (I) Quantification of relative CES1 protein levels from the immunoblot shown in (H). siCtrl, control siRNA; SP, CES1 SmartPool siRNA; A, CES1 siRNA A; B, CES1 siRNA B. Data are presented as mean ± SD (*n* = 3).

If CES1 determines cellular sensitivity to StagX1, then overexpression of CES1 in CES1-deficient cells should restore StagX1-mediated growth inhibition, whereas CES1 knockdown in CES1-positive cells should attenuate this effect. To test this, we first examined the impact of CES1 overexpression in CES1-null, StagX1-insensitive cell lines (293T, HeLa, SKNMC, and TC-71). Upon CES1 overexpression (Figure 7E), StagX1 moderately reduced cell viability in these cells (Figures 7A–7D), although the effect was less pronounced than in inherently StagX1-sensitive cell lines. This partial response may be due to transient overexpression and incomplete transfection efficiency. Next, we knocked down CES1 in the CES1-positive, StagX1-sensitive cell line COG-E-352 using siRNA. Three independent CES1 siRNAs reduced CES1 protein levels by more than 60% (Figures 7H and 7I). Notably, all three siRNAs significantly diminished the growth-inhibitory effect of StagX1 compared with control siRNA (Figure 7G). Together, these results demonstrate that CES1 is a key determinant of StagX1-mediated cell growth inhibition.

## Discussion

StagX1 belongs to the isoquinolinone derivative class and comprises an ethyl ester, an amide bond, and a 1,4-dioxane ring. All three components are speculated to contribute to the instability of StagX1.^5^ However, until now, it has remained unclear how StagX1 is metabolized and how disruption of its metabolism affects its impact on cell growth. Our research demonstrates that StagX1 is labile, and that StagX1-acid is the primary metabolite of StagX1. Our findings also suggest that ethyl ester is the main contributor to StagX1’s instability, and CES1 is responsible for hydrolyzing StagX1 into StagX1-acid. In our previous study we observed that StagX1 inhibits a set of *STAG2*-mutant ES cell lines.^3^ Additionally, according to another report, StagX1 reduces the viability of a subset of MLL cell lines with MLL/*KMT2A*-rearrangement or *CALM-AF10* translocation.^4^ However, the mechanism underlying this growth inhibition has remained unclear. Our findings suggest that StagX1-acid possesses intrinsic growth-inhibitory activity and that the observed effects of StagX1 on cell viability are likely mediated by its conversion to StagX1-acid following CES1-dependent hydrolysis.

CESs are a group of enzymes belonging to the serine hydrolase superfamily, which transform drugs, endogenous substrates, and xenobiotics containing esters, amides, carbamates, or thioesters.^7^^,^^8^^,^^9^^,^^10^^,^^11^ In humans, six CES genes have been identified, including a pseudogene. CES1 and CES2 are the most significant members for drug metabolism.^7^^,^^9^^,^^10^^,^^12^^,^^13^^,^^14^^,^^15^ These enzymes catalyze the hydrolysis of carboxylic esters into their corresponding alcohols and carboxylic acids. CES1 and CES2 have different preferences for substrate. CES1 prefers esters containing a small alcohol group and a bulky acyl group, while CES2 prefers ester substrates with a relatively large alcohol group and a small acyl group.^7^^,^^12^^,^^13^^,^^16^ StagX1 has a small alcohol group and a large acyl group, suggesting it is a preferable substrate for CES1. Our data confirms that CES1 efficiently hydrolyzes StagX1, while CES2 does not.

The tissue distribution of CES1 and CES2 enzymes differs significantly. CES1 is primarily expressed in the liver and adipose tissue, with lower levels found in kidney, lung, and intestine. On the other hand, CES2 is mainly found in the small intestine and colon, with lower expression in the liver and kidney.^7^^,^^12^^,^^16^^,^^17^ As one of the most abundant drug-metabolizing enzymes in the liver, it is not surprising that CES1 plays a crucial role in the hydrolysis of StagX1 in HLMs and mouse liver microsomes. At the cellular level, both CES1 and CES2 enzymes are located on the luminal side of the endoplasmic reticulum (ER), where they partner with other enzymes, such as UDP-glucuronosyltransferase enzymes (UGTs), to catalyze the secondary metabolism of the hydrolytic products (acids and alcohols) by attaching a glucuronic acid moiety.^7^^,^^12^ In humans, CESs is retained in cells by interacting with the KDEL (Lys-Asp-Glu-Leu) sequence of the retention protein receptor in the ER through their HXEL (His-X-Glu-Leu) motif (HIEL for CES1 and HTEL for CES2). However, in rodents, due to the absence of HXEL motif, CESs cannot be retained inside cells. As a consequence, mouse CESs are secreted into the blood, unlike human CESs that are not present in the blood.^7^^,^^8^ This may explain why StagX1 is reduced faster in mouse plasma than in human plasma in our study. Although human plasma does not have CESs, other hydrolases, such as butyrylcholinesterase, paraoxonase, and albumin esterase, etc.,^18^ can perform hydrolytic function similar to those of CESs. This may explain why StagX1 has gradually decreased in human plasma. The different tissue distribution of CES1 in mice and human suggests that the pharmacokinetic profile observed in our mouse studies may not fully reflect the metabolic fate of StagX1 in humans. Therefore, caution should be exercised when extrapolating the mouse data directly to the human setting.

Our results suggest that StagX1 can penetrate cells and is then hydrolyzed by CES1 to form StagX1-acid, which is subsequently exported and accumulated outside the cells. However, it is unclear how StagX1 enters cells or how StagX1-acid is exported and prevented from re-entering cells. Drugs and xenobiotics can enter cells through diffusion, carrier-mediated uptake, or a combination of both.^19^ Since StagX1 is a hydrophobic carboxyl ester, it is possible that it can passively diffuse across the cell membrane. However, we cannot exclude the possibility that StagX1 uptake by cells is carrier mediated. Interestingly, StagX1-acid appears to be actively exported out of the cells, as it is produced inside cells, but it is not detectable within the cells and is gradually accumulated in medium in CES1-positive SK-ES-1 cells. Moreover, StagX1-acid seems to be kept from entering cells. Hydrophobic cell membrane may repel StagX1-acid due to its negative charged acidic group after the loss of ethyl ester and becoming a hydrophilic molecule. It is also possible that cells may lack appropriate importers.

StagX1 was initially discovered as a lead molecule from a high-throughput screening process to selectively inhibit the growth of ES cells harboring mutations in the cohesin STAG2 protein.^3^ It was suggested that the sensitivity of cells to StagX1 may be linked to the status of STAG2 in those cells, with StagX1-sensitive cells being *STAG2* mutant and StagX1-insensitive cells being *STAG2* wild type.^3^ However, based on our investigation of StagX1 metabolism described here, we demonstrate that StagX1 acts as a prodrug and that its growth inhibitory activity is governed primarily by CES1 expression, rather than *STAG2* genotype.

This study presents compelling evidence to support the role of CES1 in determining the sensitivity of StagX1 in inhibiting cell growth. Firstly, StagX1 is confirmed to be a CES1 substrate. Secondly, CES1 is highly expressed in StagX1-sensitive cell lines, while no detectable levels of CES1 are found in StagX1-insensitive cell lines. Thirdly, CES1 inhibitors can block the StagX1-induced growth inhibition of cells. Finally, overexpression of CES1 can convert StagX1-insensitive cell lines into StagX1-sensitive ones, while knockdown of CES1 in StagX1-sensitive cell lines render them insensitive to StagX1. These findings collectively suggest that CES1 is the critical determinant of the sensitivity of cells to StagX1 in inhibiting cell growth.

The product of StagX1 hydrolysis by CES1 is StagX1-acid. However, it is unclear whether the inhibitory effect on cells growth is triggered by StagX1 itself or StagX1-acid. Our experiments showed that treatment with StagX1-acid alone did not result in cell growth inhibition. However, we cannot rule out that StagX1-acid has a direct role in cell growth inhibition if it can enter cells.

Public pan-cancer datasets, including TCGA and the Human Protein Atlas, indicate that CES1 expression is elevated in a subset of epithelial malignancies, such as hepatocellular carcinoma, certain gastrointestinal and lung cancers, whereas many other tumor types exhibit low or undetectable CES1 expression.^20^ However, the CES1 expression status of primary ES tumors has not been systematically characterized. Among the seven ES cell lines evaluated in this study, four expressed CES1, while three were CES1-negative.

If StagX1 was translated clinically, CES1 expression would likely serve as a predictive biomarker to identify patients most likely to benefit from StagX1 therapy. To minimize CES1-mediated hydrolysis before StagX1 reaches tumor tissue, future studies should focus on developing structurally modified analogs with improved metabolic stability and reduced susceptibility to CES1-mediated hydrolysis, as well as formulation strategies designed to protect the parent compound during systemic circulation. Our findings also suggest that StagX1 functions as a prodrug that is converted by CES1 into the active metabolite, StagX1-acid. If StagX1-acid is indeed the species responsible for the antitumor activity, the current dependence on CES1 could potentially be overcome by developing delivery strategies or structural modifications that enable efficient cellular uptake and intracellular retention of StagX1-acid. Such approaches could eliminate the need for tumor CES1 expression and substantially broaden the therapeutic applicability of this class of compounds. Together, these complementary strategies are intended to improve tumor exposure and maximize the therapeutic potential of this chemical scaffold.

In the present study, StagX1 selectively inhibited the growth of CES1-positive ES cell lines and leukemia cells, raising the possibility that it may also be effective against other CES1-positive malignancies. This hypothesis warrants further investigation in additional preclinical models representing cancers with high CES1 expression.

**Figure 8:**
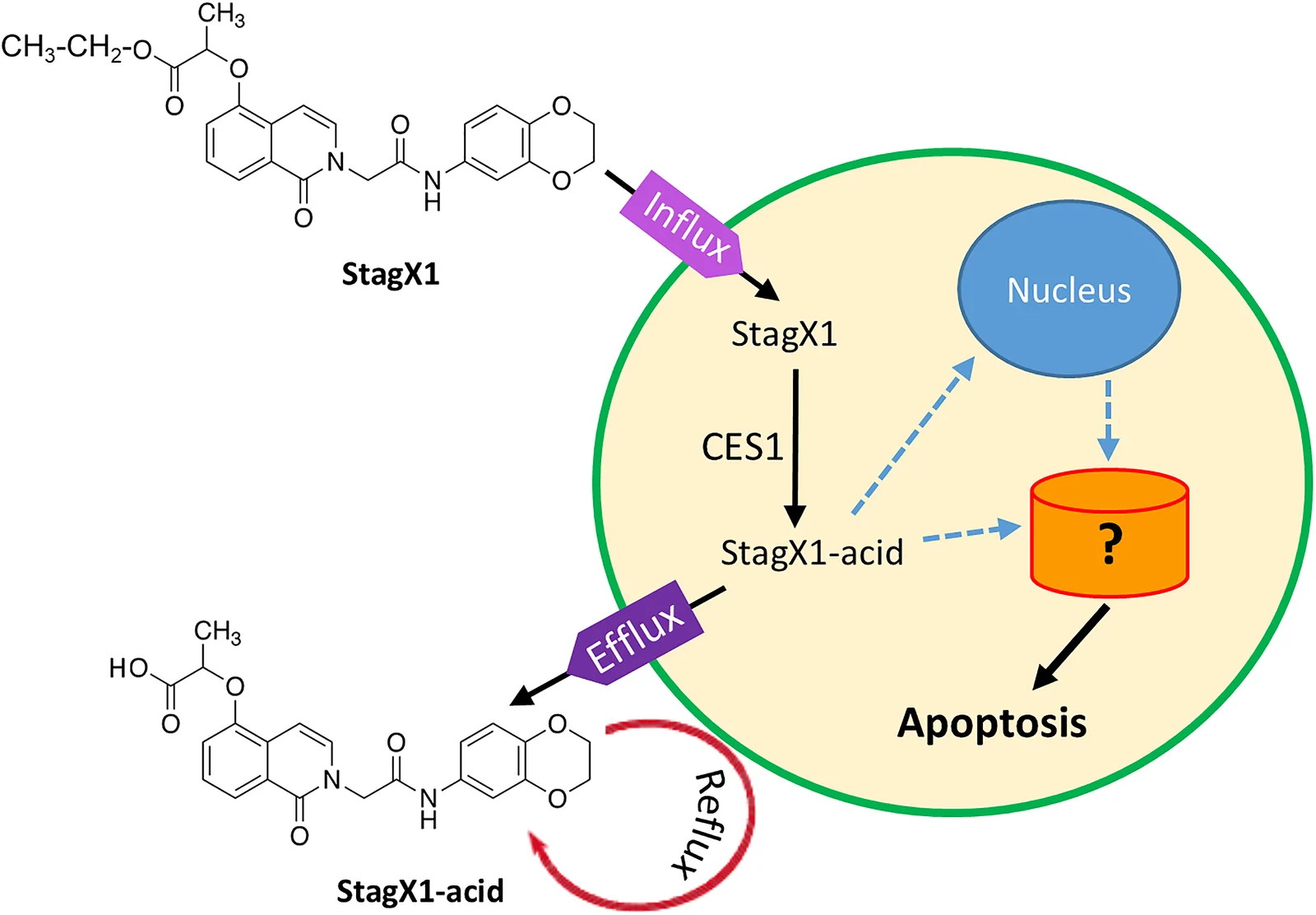
Proposed model of StagX1 metabolism and mechanism of action StagX1 enters the cells by passive diffusion and/or transporter-mediated uptake, where it is hydrolyzed by carboxylesterases (CES1) to generate Stagx1-acid. StagX1-acid is proposed to mediate the growth-inhibitory effect that leads to apoptotic cell death before being exported to the outside of the cells. It remains outside of the cells because its increased hydrophilicity prevents it crossing the plasma membrane via passive diffusion.

A summary model is presented in Figure 8. Our findings support a mechanism in which StagX1 acts as a prodrug that undergoes intracellular hydrolysis by CES1 to generate StagX1-acid. This metabolite likely initiates a downstream cascade leading to cell death before being exported from the cell. While these results clarify key aspects of StagX1 metabolism, several limitations remain. The biological activity of StagX1-acid has not yet been directly validated, and the immediate molecular targets of either StagX1 or StagX1-acid are still unknown. Despite these limitations, our study provides important insights into the metabolic fate and stability of StagX1 and establishes a foundation for the development of StagX1-derived compounds as potential therapeutic agents for ES and other malignancies.

## Materials and methods

### Materials

Nicotinamide adenine dinucleotide phosphate (NADPH) used in this study was obtained from Sigma-Aldrich (St. Louis, MO). Human/mouse liver microsomes (HLM/MLMs) and recombinant human cytochrome P450 enzymes (CYPs) (EasyCYP Bactosomes) were purchased from XenoTech (Lenexa, KS). The solvents used for liquid chromatography and mass spectrometry were of the highest commercially available grade. Antibodies used in this study were CES1 (A-11) and CES2 (G5) mouse monoclonal antibody from Santa Crus Biotechnology, Actin mouse monoclonal antibody from Sigma. SID7969543 (StagX1) was purchased from Bio-Techne (Minneapolis, MN).

### Cell culture

All cell lines used in this study were authenticated by short tandem repeat (STR) profiling and routinely tested to confirm absence of mycoplasma contamination. The cell lines A-673, SK-ES-1, SKNMC, CEM, THP1, 293T, and HeLa were obtained from ATCC (Manassas, VA). COG-E-352, TC-32, TC-71, and TC-106 were sourced from Childhood Cancer Repository (Lubbock, TX). Molm-13 were from DSMZ (Braunschweig, Germany). SK-ES-1, SKNMC, COG-E-352, TC-32, TC-71, TC-106, CEM, THP1, and Molm-13 cells were cultured in RPM1640 medium supplemented with 10% FBS. The 293T and HeLa cell lines were cultured in Dulbecco’s modified Eagle medium (DMEM) supplemented with 10% FBS. All cell lines were maintained in condition of 5% CO_2_ at 37°C. All cell lines were used within 20 passages.

### Preparation of protein samples and immunoblotting assay

Stock solution of StagX1 was prepared in DMSO. Cells were treated with 10 μM StagX1 for 0, 8, 16, and 24 h before they were harvested. Protein sample preparation and immunoblotting were performed as previously detailed.^21^^,^^22^ PARP mouse antibody (Cat# 551025, RRID:AB_394009) was from BD Biosciences (San Jose, CA). Active caspase-3 (Cat# ab32042, RRID:AB_725947) and Lamin B1 (Cat# ab16048, RRID:AB_443298) rabbit antibodies were from Abcam (Cambridge, MA). CES1 (Cat# sc-365249, RRID:AB_10850408) and CES2 (Cat# sc-100685, RRID:AB_1121519) mouse antibodies were obtained from Santa Cruz Biotechnology (Dallas, TX). β-Actin mAb (Cat# A5316, RRID:AB_476743) was bought from MilliporeSigma (Burlington, MA).

### RNA-seq

A673, COG-E-352, SK-ES-1, and TC-71 cells were treated with 10 μM STAGX1 for 0, 8, 16, 24, and 48 h. Total RNA was extracted with RNeasy Plus Mini Kit (Qiagen Cat.no. 74136). RNA-seq and analysis were performed by GENEWIZ from Azenta Life Sciences.

### Synthesis of StagX1-acid

StagX1 was synthesized following the previously reported method.^3^ To obtain StagX1-acid, the ethyl alcohol group was removed. StagX1 (1 g of ester in 3 mL of methanol) was dissolved in methanol by stirring and heated in a 35°C oil bath. Next, potassium hydroxide was added to the reaction mixture, and the mixture was allowed to stir at 35 C for 1 h. The reaction was quenched by adding additional methanol to the crude mixture. Unreacted ester was removed by extracting with diethyl ether. The carboxylic acid product was recovered by adjusting the aqueous layer to pH = 2 with 5 M hydrochloric acid. The product was precipitated, filtered, and dried under vacuum to yield StagX1-acid, which was confirmed using proton NMR (400 MHz).

### Overexpression and knockdown of CES1

The *CES1* cDNA was cloned in pCMV3-SP-N-HA vector (Sino Biological, Wayne, PA). Cells were transfected with pCMV3-SP-N-HA-*CES1* using Lipofectamine 3000 Reagent (Invitrogen) following the manufacturer’s protocol. The cells were selected with 50 mg/mL Hygromycin for three passages before being used for cell viability and immunoblotting analysis. For *CES1* knockdown, the following siRNAs were used: silencer negative control siRNA #1 siRNA (Ambion), siGEOME SMARTpool CES1 siRNA (Dharmacon), as well as custom synthesized *CES1* siRNA A (CCACCUACAUGUAUGAGdtdt) and *CES1* siRNA B (CCAUGGAGCUUUGUGAAGAdtdt). Cells were transfected with siRNA using Lipofectamine 3000 Reagent. Two days after siRNA transfection, cells were harvested for immunoblotting assay or treated with drugs for the cell viability assay.

### Metabolism of StagX1 in plasma

StagX1 was mixed with human or mouse plasma at a final concentration of 3 μM. The reaction mixtures were incubated at 37°C, and the samples are collected at specific time-points 0, 30, and 60 min. The reactions were terminated by adding equivalent volume of ice-cold methanol (containing 0.1 μM of agomelatine as the internal standard, IS) and vortexed. After centrifugation at 15,000× *g* for 15 min, the supernatant was analyzed on LC-MS.

### Metabolism of StagX1 in HLM, MLM

The incubations were carried out in 1× phosphate-buffered saline (1× PBS, pH 7.4) with 25 μM StagX1, 1.0 mg protein/mL of HLM or MLM in a final volume of 190 μL. After pre-incubation for 5 min at 37°C, the reaction was initiated by adding 10 μL of 20 mM NADPH (final concentration 1.0 mM) and continued for 30 min with shaking at 37°C. Incubations lacking NADPH served as controls. The reactions were terminated by adding 200 μL of ice-cold methanol, followed by vortexing for 30 s, and centrifuging at 15,000× *g* for 15 min. The supernatant was used for StagX1 and StagX1-acid analysis on LC-MS.

### Identification of StagX1-acid

StagX1-acid was identified using the exact mass and synthetic standard compound.

### Pharmacokinetic study of StagX1 in mice

SCID-beige mice were obtained from Inotiv (Indianapolis, Indiana). All animal experiments were approved by the Institutional Animal Care and Use Committee of Baylor College of Medicine (protocol number AN-4672), and the animal studies were carried out in strict compliance with federal and local guidelines for animal care and use. Seven to eight weeks old SCID-beige mice were used, with 4 mice (2 male and 2 female) per group. StagX1 was diluted in PBS at a concentration of 100 μM and administered via intraperitoneal injection at a dose of 450 μg per kilogram of body weight. Blood, liver, and kidney samples were collected at 3, 15, and 30 min, as well as 1, 2, 4, 8, and 24 h after injection. Blood was collected using micro sample tube K3 EDTA (Sarstedt Inc., Germany), and plasma was separated by centrifugation at 2,000× *g* for 8 min. Liver and one kidney samples were harvested, washed with PBS three times, weighed, and frozen in dry ice.

The plasma samples were prepared by mixing 10 μL of plasma with 60 μL of ice-cold methanol (containing 0.1 μM agomelatine as the internal standard, IS).^23^ The sample mixtures were vortexed, centrifuged at 15,000× *g* for 15 min. Liver and kidney samples were weighed and homogenized in 50% methanol with the ratios of 1:10 and 1:6 (w/v), respectively. To 50 μL of the liver or kidney homogenate, 200 μL of IS solution was added, and the mixtures were vortexed, centrifuged at 15,000× *g* for 15 min. The resulting supernatants were transferred to a new Eppendorf tube and subjected to a second centrifugation at 15,000× *g* for 15 min. Three microliters of each supernatant were injected for ultra-high performance liquid chromatography coupled with a mass spectrometry (UHPLC-MS) analysis.

To quantify the StagX1 and StagX1-acid in samples, standard curves were generated. The StagX1 and StagX1-acid are serially diluted with methanol to obtain work solutions with concentration range of 10 nM–100 μM. Five microliters of the work solutions were spiked to 5 μL of mouse plasma, kidney, or liver homogenate, followed by adding 30 μL of methanol (containing 0.1 μM of agomelatine as IS) and processed in the same way as the plasma samples described previously. The final linear ranges for the standard curve are 10 nM–100 μM (according to the concentration ranges obtained from the samples) with the regression weight of 1/×^2^. The lower limit of quantification (LLOQ) for StagX1 was 0.452 ng/mL and for StagX1-acid was 2.125 ng/mL.

All the samples were analyzed using a Vanquish UHPLC coupled with a Q Exactive Hybrid Quadrupole-Orbitrap MS (Q Exactive MS) (Thermo Fisher Scientific, San Jose, CA). Q Exactive MS was operated in positive mode with electrospray ionization. For full MS, data ranging from m/z 80 to 1,200 Da in-profile mode was acquired. The ion at m/z 371.1012 was used as a reference ion for positive mode during acquisition. The mass resolution for full MS was 140,000.

PK parameters were estimated using PKSolver with an extravascular non-compartmental model.^24^ Nominal sampling time is relative to the start of each dose administration.

### CES1 and CES2 hydrolyzing StagX1 assay

Twenty nanogram of recombinant human CES1 or CES2 protein (Bio-techne, Minneapolis, MN) were mixed with StagX1 (final concentration was 3 μM) in 50 μL of assay buffer (50 mM Tris-HCl, pH 7.5). The reaction mixture was incubated 22°C for 0, 5, 15, and 30 min in triplicate. To investigate the inhibition of CES1 enzymatic activity by benzil, CES1 and benzil were incubated for 5 min before StagX1 was added. The concentration of benzil in the reaction mixture was 4 μM. To stop the reaction, 100 μL of ice-cold MeOH was added and vortexed for 30 s. After centrifugation at 15,000× *g* for 15 min, the supernatant was collected for LC-MS analysis.

### Data analysis

The LC-MS data was acquired with the Xcalibur software (Thermo Fisher Scientific, San Jose, CA).

### Preparation of medium and cell samples for StagX1 and StagX1-acid analysis

Cells were seeded at a density of 2 × 10^5^ cells per well in 6-well plates and incubated for 48 h. The culture medium was replaced with fresh medium, with or without 5 μM of benzil. After 3 h, the cells were treated with 5 μM StagX1 for different time intervals, including 0, 15, 30, 60 min, and 24 h. At each time point, 0.4 mL medium was collected. To collect the cells, the medium in each well was removed, and the cells were rinsed with 2 × 2 mL of PBS. The cells were then scraped and suspended in 0.4 mL PBS. To extract StagX1 and StagX1-acid from the samples, 0.8 mL of cold methanol was added to the medium or the cell suspension, and the samples were vortexed for 30 s. The samples were then centrifuged at 15,000× *g* for 15 min, and the supernatant was collected for analysis using LC-MS.

### Cell viability assay

The cell viability assay was conducted as previously described.^25^ Cells were seeded onto 96-well plates in medium containing 10% FBS and incubated overnight at 37°C, 5% CO_2_. After 72 h of treatment with various concentration of drugs, viability of the cells was determined using CellTiter-Blue Reagent (Promega, Madison WI). Briefly, the CellTiter-Blue Reagent was added to each well, and the plates were incubated for an additional 6 h at 37°C with 5% CO_2_. The fluorescence of the samples was then measured using a fluorescence plate reader, and the results were normalized to the untreated control cells. The viability of the cells was calculated as a percentage of the control cells.

## Data and code availability

The raw data supporting the conclusions of this article will be made available by the authors on request.

## Acknowledgments

This study was made possible through funding provided by the Cancer Prevention and Research Institute of Texas (CPRIT) grant RP190002 as well as a grant from the Kate Amato Foundation awarded to D.P.

## Author contributions

Conceptualization, D.P. and N.Z.; methodology, N.Z., F.L., S.R.G., and D.P.; bioinformatics data analysis, N.Z.; validation, N.Z. and F.L.; formal analysis, N.Z., F.L., and D.P.; investigation, N.Z. and F.L.; resources, D.P., F.L., and S.R.G.; data curation, N.Z.; writing – original draft preparation, N.Z., writing – review and editing, D.P., F.L., and S.R.G.; visualization, N.Z. and F.L.; supervision, D.P.; project administration, D.P.; funding acquisition, D.P.

## Declaration of interests

The authors declare no competing interests.

## Supplemental information

Document S1. Figures S1–S3 and Table S1

Document S2. Article plus supplemental information
