# Supplementary material for: StagX1, an isoquinolinone compound, selectively targets CES1-positive Ewing sarcoma cells as a potential therapeutic agent
Source: Mol Ther Oncol. 2026 Aug 11;34(3):201321. doi: 10.1016/j.omton.2026.201321 (PMC13524716; doi:10.1016/j.omton.2026.201321)
Supplement: Supplementary file 1 [file mmc1.pdf]

OMTON, Volume *34*

Supplemental information

StagX1, an isoquinolinone compound, selectively targets CES1-positive Ewing sarcoma cells as a potential therapeutic agent

Nenggang Zhang, Scott R. Gilbertson, Feng Li, and Debananda Pati

Table S1. Mean summary of pharmacokinetic analysis of StagX1 and StagX1-acid in mice.

Parameter	Plasma		Liver		Kidney	
	StagX1	StagX1-acid	StagX1	StagX1-acid	StagX1	StagX1-acid
Conc. Unit	μg/ml	μg/ml	μg/g	μg/g	μg/g	μg/g
Lambda_z (1/min)	N/A	0.018	0.0003	0.003	N/A	0.003
$t_{1/2}$ (min)	N/A	39.063	2265.691	203.061	N/A	234.345
T_{max} (min)	N/A	15	3	30	N/A	15
C_{max}	N/A	25.705	0.032	1.030	N/A	1.241
Tlag (min)	N/A	0	0	0	N/A	0
Clast_obs/Cmax	N/A	0.022	0.085	0.003	N/A	0.010
AUC_{0-t} (conc*min)	N/A	2156.802	6.041	71.449	N/A	130.722
AUC_{0-inf}_obs (conc*min)	N/A	2188.530	15.083	72.316	N/A	134.934
$AUC_{0-t/0-inf}$_obs	N/A	0.986	0.401	0.988	N/A	0.969
$AUMC_{0-inf}$_obs (conc*min^2)	N/A	135306.225	45913.666	8296.878	N/A	29441.399
MRT_{0-inf}_obs (min)	N/A	61.825	3043.974	114.731	N/A	218.190
Vz/F_obs (μg/conc)	N/A	11.588	178783.795	1822.976	N/A	1127.509
Cl/F_obs (μg/conc/min)	N/A	0.206	54.696	6.223	N/A	3.335
StagX1/StagX1-acid C_{max} ratio	N/A		0.032		N/A	

N/A: not available due to beyond LLOQ of StagX1. Data represents mean of 4 mice.

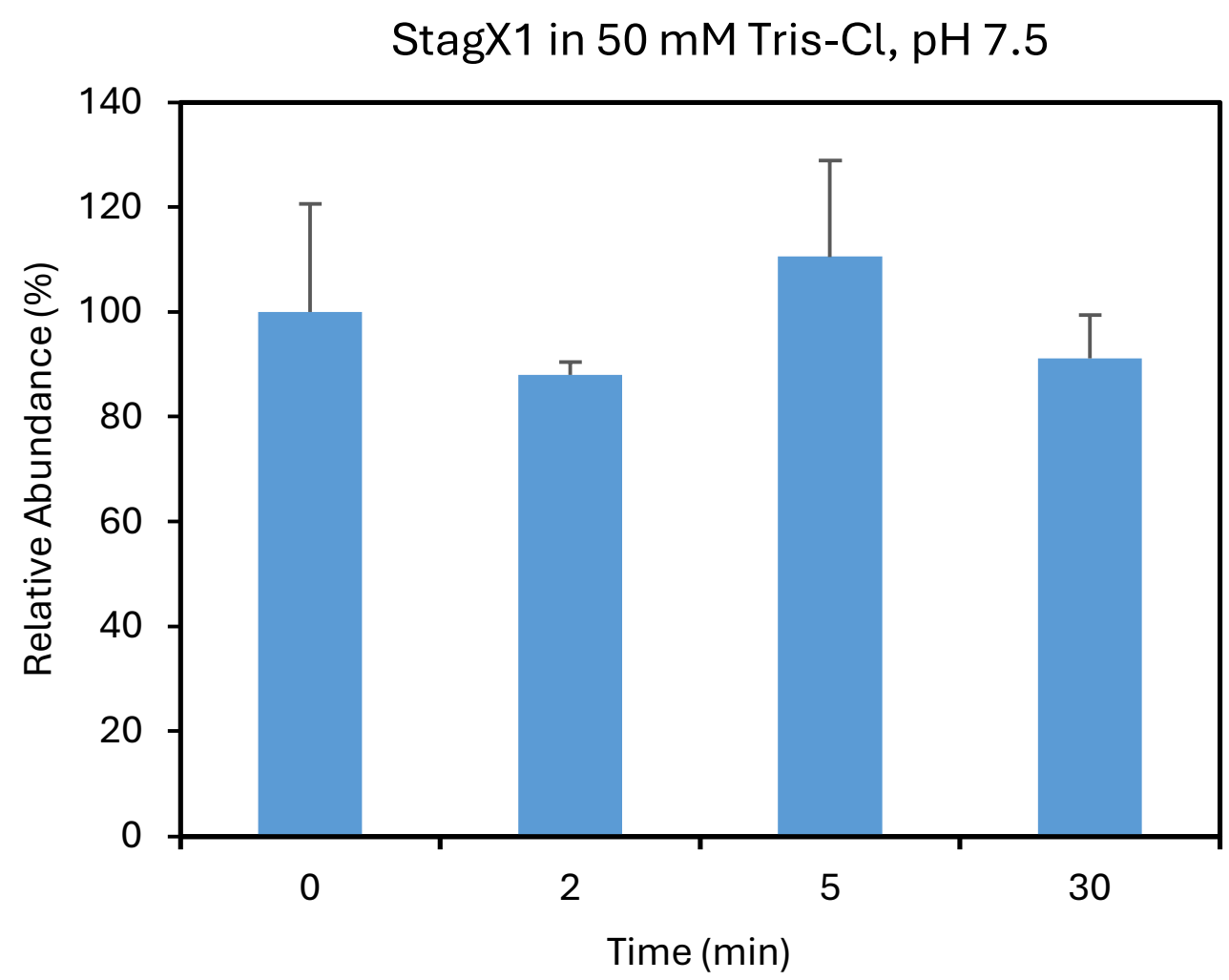

Figure S1. Stability of StagX1 in CES1 assay buffer. StagX1 was incubated in assay buffer (50 mM Tris-Cl, pH 7.5) in the absence of enzyme, and samples were collected at indicated time points to determine StagX1 concentration by LC-MS. The results demonstrate that StagX1 is stable under the assay conditions. Data are presented as mean ± SD (n=3).

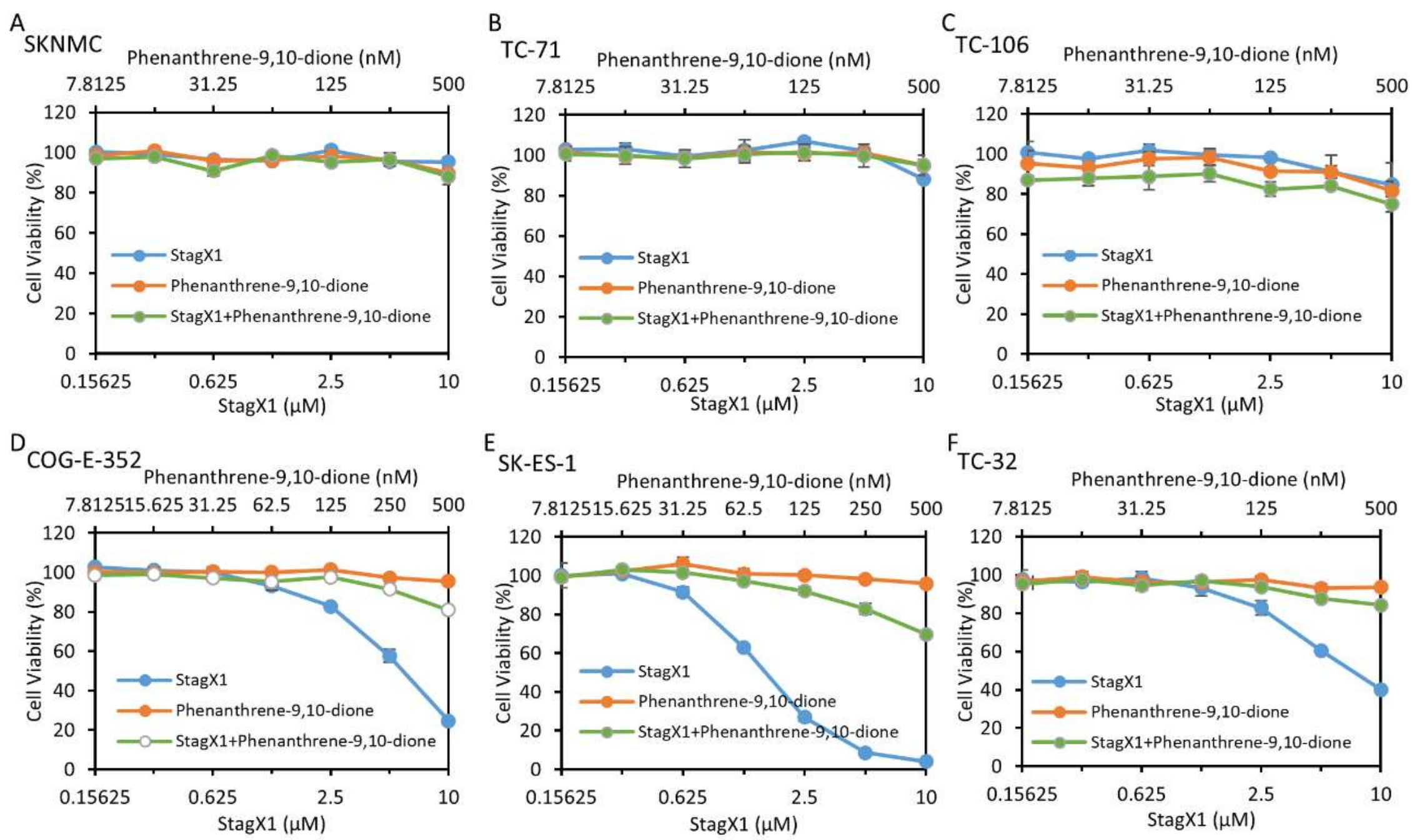

Figure S2. The CES inhibitor Phenanthrene-9,10-dione blocks the growth-inhibitory effect of StagX1. Treatment with Phenanthrene-9,10-dione, StagX1, or their combination had no effect on the growth of the CES1-negative Ewing sarcoma cell lines SKNMC, TC71, and TC106 (A-C). In contrast, Phenanthrene-9,10-dione abolished the growth-inhibitory effect of StagX1 in the CES1-positive Ewing sarcoma cell lines COG-E-352, SKES-1, and TC-32 (D-F). Data are presented as mean ± SD (n=3).

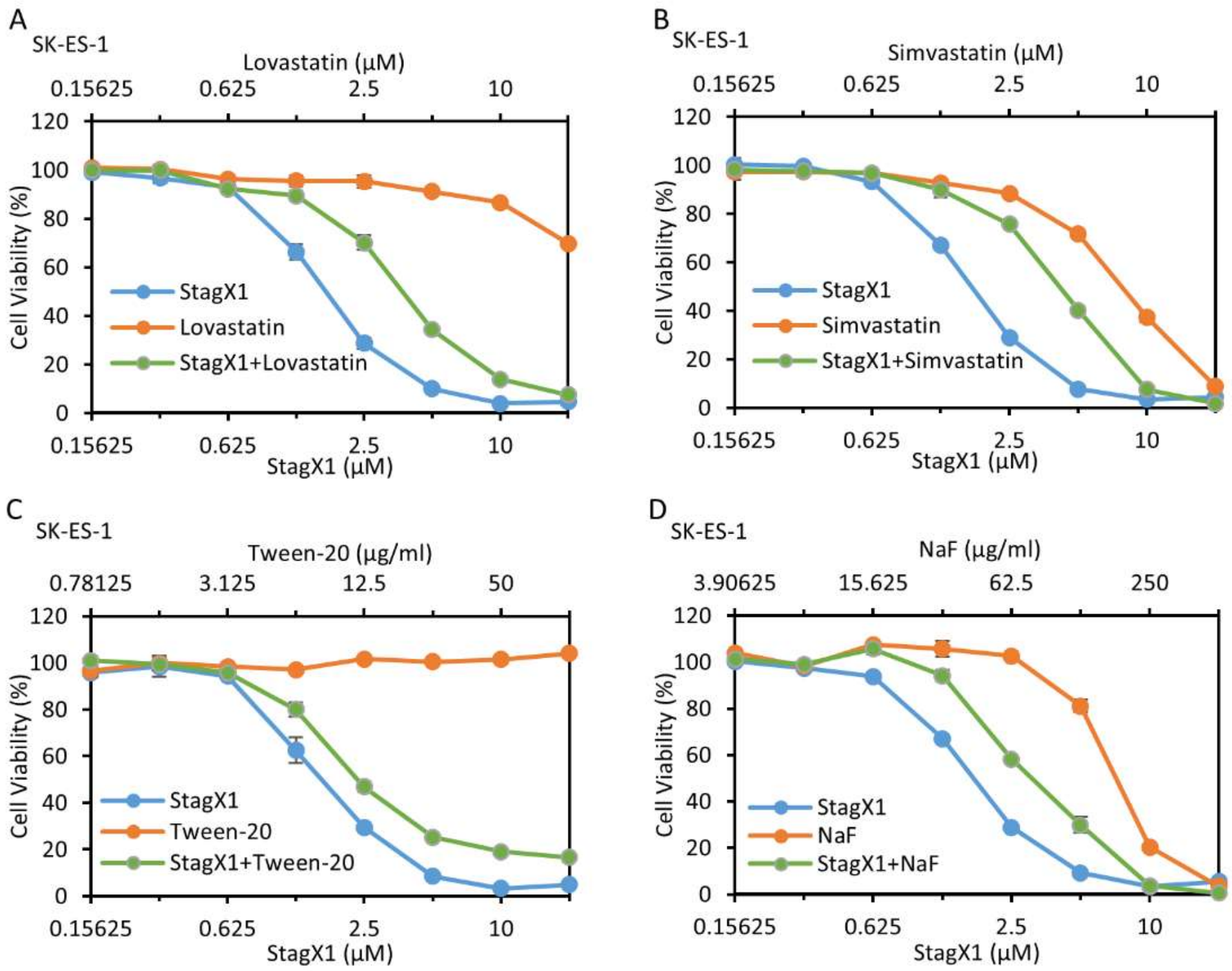

Figure S3. Effects of CES inhibitors on the growth-inhibitory activity of StagX1 in SK-ES-1 cells. The effects of various CES inhibitors on StagX1-mediated growth inhibition were evaluated in the CES1-posiitive Ewing sarcoma cell line SK-ES-1. Lovastatin (A), Simvastatin (B), Tween-20 (C), and Sodium Fluoride (NaF) (D) attenuated the inhibitory effect of StagX1. Data are presented as mean ± SD (n = 3).
